# Candida Sepsis Following Transcervical Chorionic Villi Sampling

**DOI:** 10.1155/S1064744901000266

**Published:** 2001

**Authors:** Alona Paz, Roni Gonen, Israel Potasman

**Affiliations:** 1 Infectious Diseases Bnai Zion Medical Center PO Box 4940 47 Golomb St. Haifa 31048 Israel; 2 Department of Obstetrics and Gynecology Bnai Zion Medical Center Rappaport Faculty of Medicine Technion Haifa Israel

## Abstract

*Background:* The use of invasive devices and broad spectrum antibiotics has increased the rate of candidal superinfections.Candida sepsis associated with pregnancy is rare. Candida sepsis following chorionic villi sampling (CVS) has never been reported.

*Case:* A 31-year-old pregnant woman presented with signs of sepsis one day after undergoing transcervical CVS. Blood culture and curettage material yielded *C. albicans. * She was treated with 400 mg of fluconazole daily for 4 weeks and completely recovered.

*Conclusion:* Candida sepsis should be considered in the differential diagnosis of sepsis following CVS.


Infect Dis Obstet Gynecol 2001;9:147–148

### Candida sepsis following transcervical chorionic villi sampling

**Alona Paz[1], Roni Gonen[2] and Israel Potasman[1]**

[1]*Infectious Diseases, Bnai Zion Medical Center*
[2]*Department of Obstetrics and Gynecology, Bnai Zion Medical Center, Rappaport Faculty of Medicine, Technion, Haifa, Israel*

***Background:*** The use of invasive devices and broad spectrum antibiotics has increased the rate of candidal superinfections. Candida sepsis associated with pregnancy is rare. Candida sepsis following chorionic villi sampling (CVS) has never been reported.
***Case:*** A 31-year-old pregnant woman presented with signs of sepsis one day after undergoing transcervical CVS. Blood culture and curettage material yielded *C. albicans*. She was treated with 400 mg of fluconazole daily for 4 weeks and completely recovered.
***Conclusion:*** Candida sepsis should be considered in the differential diagnosis of sepsis following CVS.

Key words: CANDIDA SEPSIS; PREGNANCY; CHORIONIC VILLI SAMPLING; FLUCONAZOLE

Fungal infections constitute an important cause of morbidity and mortality. Over the past decade, the use of invasive devices and the administration of broad-spectrum antimicrobial agents have resulted in an increase in the incidence of these infections[1]. Candidemia is associated with an attributable mortality of 38% and prolongation of hospital stay by as much as 30 days[2]. Pregnant women have an increased rate of vaginal colonization with *Candida* spp.[3]. Nevertheless, only a few cases of Candida sepsis associated with pregnancy have been documented[4]. Chorionic villi sampling (CVS) is considered a relatively safe procedure, although an invasive one. Uterine infections after CVS pose an uncommon, albeit severe complication that can lead to maternal sepsis[5]. Candida sepsis following CVS has never been reported. We hereby report a case of septic abortion due to *Candida albicans* following CVS.

### CASE REPORT

A 31-year-old woman, gravida 3, following two normal deliveries, was admitted to the hospital with suspected septic abortion in her 10th week of pregnancy, 1 day after having undergone transcervical CVS. The CVS was performed as per the patient's wish. The patient complained of fever, chills, abdominal pain and blood-stained vaginal discharge. Her temperature on admittance was 38.5°C and her abdomen was diffusely tender, without vaginal bleeding. Her white blood cell count (WBC) was 9500 cells/μl on admittance. A fetal heart beat was detected by ultrasonography. As infection was suspected, empiric antibiotic therapy with ampicillin, gentamicin and metronidazole was initiated. Since her clinical condition deteriorated, with temperature spiking to 39°C and WBC to 22 000 cells/μl, aspiration curretage was performed 12 h after her admission. The

Correspondence to: Israel Potasman, MD, Infectious Diseases, Bnai Zion Medical Center, PO Box 4940, 47 Golomb St., Haifa 31048, Israel. Email: Ipotasma@netvision.net.il evacuated material was sent for culture and histologic examination. Following the procedure, her temperature dropped, as did her WBC (down to 5000), but the woman kept complaining of severe headache. A total of six blood cultures were taken during the first 3 days of her hospitalization. One day after admission, one blood culture and a culture of the evacuated material yielded *C. albicans*. Because of the fungemia and the persistent headache and vomiting, CNS involvement was suspected. However, repeated neurologic examinations, fundoscopy, lumbar puncture, computed tomography of the brain and electroencephalography gave results all within normal ranges. Antibiotic administration was discontinued, and treatment with fluconazole 400 mg daily was initiated. The pathologic examination revealed an inflamed decidua with secretory endometrial glands, placental villi and blood clots. Histopathologic sections stained for fungi (Grocot and PAS stains) were negative, although cultures were positive as mentioned above. Repeated blood cultures taken 2 days after initiation of treatment were negative. Her headache gradually subsided and she was subsequently treated with fluconazole for 4 weeks.

### DISCUSSION

Infectious complications after CVS are rare. Brambati and colleagues[6] found uterine infection to occur in only 0.13% of patients following transcervical CVS. Fulminant sepsis due to group B β-hemolytic streptococcus is one of the well-documented examples[7]. Becker and co-workers[8] found a significantly higher risk of abortion (relative risk 8.9) following transcervical CVS in connection with one microorganism or more, isolated from the cervical canal before the procedure. Candida is a well-recognized colonizer of the vagina. During pregnancy, the rate of vaginal colonization with *Candida* spp. increases to 30%. It is conceivable, therefore, that candida sepsis would occur during pregnancy or invasive procedures related to pregnancy. In fact, it is surprising that in a recent review we found only eight cases of candida sepsis during pregnancy[4]. These cases were mostly related to risk factors such as foreign bodies (intrauterine device) and antibiotic therapy. The present article is the first reported case of the development of an invasive systemic candidal infection following CVS. Infection was probably disseminated during the procedure, as evidenced by the growth of candida from the product of conception. The absence of candida in the pathologic sections may be due to a sampling error. This case raises the question of whether vaginal colonization by candida should be considered a risk factor for infection following transcervical CVS, and whether it should be examined and treated prior to this procedure in order to avoid the above described serious complications. For all practical purposes, we recommend a high index of suspicion in women who develop sepsis following CVS.

### REFERENCES

1. Jarvis WR. Epidemiology of nosocomial fungal infections, with emphasis on candida species. *Clin Infect Dis* 1995;20:1526–30
2. Uzun O, Anaissie EJ. Problems and controversies in the management of hematogenous candidiasis. *Clin Infect Dis* 1996;22(Suppl 2):S95–101
3. Fleury FJ. Adult vaginitis. *Clin Obstet Gynecol* 1981; 24:407–38
4. Potasman I, Leibovitz Z, Sharf M. Candida sepsis in pregnancy and the post partum period. *Rev Infect Dis* 1991;13:146–9
5. Jauniaux E, Rodeck C. Use, risks and complications of amniocentesis and chorionic villus sampling for prenatal diagnosis in early pregnancy. *Early Pregnancy* 1995;1:245–52
6. Brambati B, Lanzani A, Tului L. Transabdominal and transcervical CVS: efficiency and risk evaluation of 2144 cases. *Am J Med Genet* 1990;35:160–4
7. Fejgin M, Amiel A, Kaneti H, *et al.* Fulminant sepsis due to group B beta-hemolytic streptococci following transcervical chorionic villi sampling. *Clin Infect Dis* 1993;17:142–3
8. Becker R, Mende B, Rodloff AC, *et al.* Bacteriologic findings before and in transcervical chorionic villi biopsy and their clinical relevance. *Geburtshilfe Frauenheilkd* 1991;51:704–9

RECEIVED 5/31/00; ACCEPTED 5/1/01
